# The roles of self-compassion and social support on the maternal adjustment to a child’s hip dysplasia

**DOI:** 10.1177/13591053241295892

**Published:** 2024-11-21

**Authors:** Bruna Veloso, Lara Palmeira, Lénia Carvalhais, Joana Marta-Simões, Inês A Trindade

**Affiliations:** 1 Portucalense University, Portugal; 2 University of Coimbra, Portugal; 3 Örebro University, Sweden; 4 Life Quality Research Centre (CIEQV), Portugal

**Keywords:** chronic illness in children, hip dysplasia, maternal adjustment, self-compassion, social support

## Abstract

Parenthood can be challenging when facing a child’s chronic illness such as developmental dysplasia of the hip (DDH). Although social support is known as a protective factor for the caregiver’s mental health, the role of self-compassion is less explored. This study, conducted in Portugal, explored whether self-compassion and social support mediate the relationship between mothers’ psychological adjustment and perception of their child’s illness. Ninety-four mothers of children with DDH completed questionnaires on illness perception, self-compassion, perceived social support, and psychological distress. Results suggested that self-compassion and social support mediated the relationship between mothers’ overall negative perception of the children’s illness and psychological distress. The final model accounted for 50% of the variance of depressive symptoms, 40% of anxiety, and 63% of perceived stress. This study highlights the potential value of encouraging mothers to seek social support when facing their child’s DDH diagnosis. Promoting self-compassion may be important in clinical intervention.

## Introduction

Parenthood can be challenging, more so when parents are confronted with the diagnosis of a child’s chronic illness (CI) such as developmental dysplasia of the hip (DDH), a common hip disorder in children (Gibbard et al., 2021). Literature highlights that parents of children with a CI often endure significant worries associated with their child’s health and well-being (Cousineau et al., 2019). Hence, these parents may experience some negative outcomes, such as poor mental health (e.g. Cohn et al., 2020). In a systematic review, Cousino and Hazen (2013) found an association between greater parenting stress and poorer psychological adjustment in caregivers of children with CI. This may be particularly relevant for mothers, who are still seen as the primary caregivers of children with a CI, despite the growing involvement of fathers in the caregiving role (Spurr et al., 2023).

DDH is usually detected in the neonatal period and encompasses a wide spectrum of clinical severity (e.g. Gibbard et al., 2021). Known risk factors include incorrect lower-extremity swaddling, breech position, female sex, and positive family history (Morello et al., 2023). Even though the treatment for DDH varies according to certain factors (e.g. condition severity, age at diagnosis, professional opinion), the use of a harness is the most common mode of treatment (Gibbard et al., 2021). If untreated, DDH is one of the main causes of disability in childhood (Gyurkovits et al., 2021). Some possible consequences are the development of a limp, limb length discrepancy, pain, and early development of osteoarthritis (Yang et al., 2019). The diagnosis and treatment of DDH have been associated with negative psychological consequences for parents (e.g. Theunissen et al., 2022) and a recent study by Gibbard et al. (2021) suggested that DDH can be a significant burden on caregivers. Furthermore, Wakely et al. (2021) found that having a child with DDH impacted most areas of parenting, not only emotionally but also with practicalities (e.g. difficulties with feeding and settling the baby to sleep).

According to the common sense model, people’s perceptions regarding their illness (e.g. causes, controllability, severity, duration, and uncertainty) can influence their coping responses and even illness outcomes such as adjustment and illness progression (e.g. Leventhal et al., 2016). Overall, studies across multiple diseases and populations (for a meta-analysis see Hagger et al., 2017) consistently demonstrate that while negative illness perceptions and emotional representations are related to avoidant coping and worse outcomes (namely more negative emotional responses, lower quality of life, and poorer functioning), positive perceptions are linked with more adaptive coping skills, better functioning, and reduced distress. Although less studied, parents’ negative illness cognitions of their child’s illness (e.g. cognitions regarding how stressful and life-threatening the illness is to the child, the intensity of treatment, and the ability to cope) are associated with increased general parent distress and parenting-specific stress (e.g. Chaney et al., 2016) and may also negatively influence the child’s adjustment and the psychological adjustment of the entire family (Mullins et al., 2016). Therefore, it seems crucial to identify inter and intrapersonal factors that can positively impact parents’ mental health.

According to Yang et al. (2022), social support is a protective factor for the mental health of caregivers of children with CI. Social support is usually defined as the social resources individuals perceive to be available or receive, from non-professional sources, both formally and informally (Gottlieb and Bergen, 2010). For instance, in their qualitative study, Baker and Claridge (2022) demonstrated that the support received from extended family and doctors played an important role in the family’s ability to overcome stressors associated with CI. Likewise, a study with caregivers of children with CI (Toledano-Toledano and Luna, 2020) found that the perception of adequate family support can boost resilience and well-being, as well as reduce levels of anxiety, depression, burden, and parental stress.

In addition to social support, self-compassion is one of the psychological processes that has raised growing interest as a protective factor against maladjustment (e.g. Ewert et al., 2021). Self-compassion involves acknowledging and accepting one’s suffering instead of trying to ignore or disconnect from it (Neff, 2003). It entails self-kindness (versus self-judgment), common humanity (versus isolation), and mindfulness (versus overidentification) (Neff, 2023). Self-compassion has been associated with several positive outcomes (for a meta-analysis see Ferrari et al., 2019), including increasing the capacity for connection, which is particularly relevant for parents with CI children (Neff and Faso, 2014). In fact, in the context of CI, it has also proven to be a powerful tool, for example, (i) among patients with lung cancer, associations have been found between greater levels of self-compassion and less severity of shame feelings and depressive symptoms (Siwik et al., 2022), (ii) self-compassion was linked to lower levels of distress and greater well-being in a sample of adolescents and young adults with CI (Prentice et al., 2021), (iii) in a longitudinal study with adults with inflammatory bowel disease, self-compassion predicted lower subsequent levels of depressive, anxiety, and stress symptoms (Trindade and Sirois, 2021). Specifically, research on the role of self-compassion skills among caregivers of children with CI is still scarce. Worth mentioning, a study with parents of children with autism revealed that parents with higher levels of self-compassion tend to present greater life satisfaction and hope and less depression and parental stress (Neff and Faso, 2014). Within this context, self-compassion may also be a potential resilience factor regarding the risk of burnout in parental caregiving (e.g. Gerber et al., 2021) and could be useful in relieving enduring despair, hopelessness, and chronic weariness (Cousineau et al., 2019). Finally, recent compassion-based intervention studies with parents of children with CI (Ahmed and Raj, 2023; Khosrobeigi et al., 2022) showed that these interventions can improve resilience, optimism and well-being and decrease psychopathological symptoms.

Despite previous findings regarding the possible negative consequences a child’s diagnosis of CI can have on parents, there is still a lack of studies focused on factors that can have a positive impact on the adjustment of mothers with a child with a CI. Moreover, as far as we know, no studies have explored the potential protective role of inter and intrapersonal in mothers of children with DDH. Exploring parental resources in mothers of children with a common hip illness such as DDH may be particularly relevant as different CI illnesses pose distinct and unique caregiver challenges (Cousineau et al., 2019). Thus, this study has two main objectives. On the one hand, we aim to understand the relationship between mothers’ perceptions of their child’s DDH, self-compassion, social support, and psychological distress. On the other hand, we aim to explore the mediating role of inter and intrapersonal factors (social support and self-compassion) of mothers in the relationship between their perception of their children’s DDH and their psychological adjustment. We hypothesize that (i) negative illness perceptions are related to higher severity of psychological distress, and lower levels of self-compassion and social support; (ii) social support and self-compassion explain the relationship between mother’s perception of their children’s DDH and their psychological adjustment.

## Materials and methods

### Procedures

The study received approval from the Faculty of Psychology and Social Sciences of Coimbra’s University Ethics Committee before data collection. Data were collected online, through Facebook and Instagram advertisements. Additionally, emails were sent to institutions that support parents of children with chronic illnesses. The inclusion criteria to participate in this study were as follows: (a) being the mother of a child with a diagnosis of DDH established during the previous 12 months; (b) being 18 years old or older; (c) being fluent in Portuguese; (d) having internet access. Participants were excluded if they did not meet all the above criteria. The questionnaire was distributed via a Qualtrics link that provided information concerning the main objectives and the ethical considerations issues related to the study (e.g. voluntary participation, guarantee of confidentiality and anonymity). Those who provided informed consent, by clicking the “accept to participate” button, had full access to the protocol and were invited to complete it. Overall, 126 mothers accepted to participate in the study, with 32 of those (25.4%) not completing at least one of the measures of the research protocol and thus being excluded.

### Participants

The sample comprised 94 women, aged between 25 and 44 years (*M* = 34.41; *SD* = 4.06), who had a child with the diagnosis of DDH. While 91.5% (*n* = 86) of participants were married or living in a de facto partnership, 7.4% (*n* = 7) were single and 1 was widowed. The majority completed higher education (*n* = 68, 72.3%). Participants had between one and three children (*M* = 1.70; *SD* = 0.77).

Regarding children with the diagnosis of DDH, 54.3% were females (*n* = 51) and 45.7% (*n* = 43) males. Their ages varied between a few days (less than a month) and 6 years (*M* = 17.80 months; *SD* = 17.82). The two most common treatments for DDH involved using a harness (50%) and surgery (21.3%).

### Measures

#### Sociodemographic questionnaire

This self-report questionnaire gathered information regarding the sociodemographic characteristics of the mothers (e.g. age, marital status, educational level, number of children), and their child’s with DDH (e.g. gender, age, type of treatment).

#### Brief Illness Perception Questionnaire (BRIEF-IPQ; Broadbent et al., 2006, Portuguese version (PV) by Figueiras et al., 2010)

This questionnaire evaluates illness perception and comprises nine items. The first eight are answered on an 11-point Likert scale, ranging from 0 to 10, regarding consequences, timeline, personal control, treatment control, identity, concern, understanding, and emotional response. The ninth item is an open question that asks patients to list the three main causal factors in their illness. Higher scores indicate more threatening illness perceptions (understanding, personal, and treatment control are inversely scored). For the present study, instructions were slightly modified to assess mothers’ perception of their children’s DDH, for example, “How much does your baby’s illness affect your life?” instead of “How much does your illness affect your life?”. In our sample, this adapted scale’s Cronbach’s alpha was 0.75.

#### Self-Compassion Scale—Short Form (SCS-SF; Raes et al., 2011; PV by Castilho et al., 2015)

This scale includes 12 items, using a 5-point Likert scale, that ranges from 1 (*almost never*) to 5 (*almost always*) and has the following subscales: self-kindness, self-judgment, common humanity, isolation, mindfulness, and overidentification. Some of the items are reverse-scored so higher total scores indicate more self-compassion. In the Portuguese version of SCS-SF, Cronbach’s alpha for the total scale was 0.89 for a non-clinical sample. In our sample, Cronbach’s alpha was 0.83.

#### Multidimensional Scale of Perceived Social Support (MSPSS; Zimet et al., 1988; PV by Carvalho et al., 2011)

It includes 12 items, answered on a 7-point Likert scale, ranging from 1 (*very strongly disagree*) to 7 (*very strongly agree*). It encompasses three dimensions: family, friends, and significant others. The higher the score, the higher the perceived social support. Cronbach’s alpha was 0.94 both in the Portuguese version and our sample.

#### Perceived Stress Scale-10 (PSS-10; Cohen et al., 1983; PV by Trigo et al., 2010)

This questionnaire aims to assess stress levels in the month before completion. It comprises 10 items, answered on a 5-point Likert scale ranging from 0 (*never*) to 4 (*very often*). Higher scores represent higher levels of stress. In the Portuguese version of PSS-10, Cronbach’s alpha was 0.87 while in our sample it was 0.89.

#### Hospital Anxiety and Depression Scale (HADS; Zigmond and Snaith, 1983; PV by Pais-Ribeiro et al., 2007)

This questionnaire was used to assess the level of anxiety or depressive symptoms in the week before completion. It comprises two subscales, with seven items each, answered on a 4-point Likert scale, ranging from 0 to 3. Higher scores indicate higher psychological distress. The Portuguese version of HADS obtained alphas of 0.81 and 0.76 for the depression and anxiety subscales, respectively. Similarly, in our sample, the Cronbach’s alphas were 0.81 and 0.80.

### Data analyses

Data analyses were performed using IBM SPSS Statistics, version 27.0 (descriptive and correlational analyses) and AMOS software for the path analyses (structural equation models). Power analysis was calculated a *priori* using G*Power 3.1 for a Multiple Regression Analysis with three predictors. Results indicated that a sample size of 90 was needed, using a significance level of 0.05, a power of 95%, with a medium effect size (*f* = 0.20).

Demographic and DDH data were examined through descriptive analyses. The mean and standard deviation scores of all variables in the study were also examined through descriptive analyses. To evaluate the relationships between the different variables under study Pearson correlations were performed. The interpretation of these correlations was made following Cohen (1988): *r* values between 0.10 and 0.29 are considered weak correlations, between 0.30 and 0.49 moderate, and between 0.50 and 1 strong.

A path analysis was performed to explore the mediating roles of self-compassion (SCS-SF) and social support (MSPSS) in the relationship between mothers’ perception of their child’s DDH and psychological distress (HADS and PSS-10). Path analysis allows the simultaneous examination of structural relationships, as well as the examination of direct and indirect paths (e.g. Schumacker and Lomax, 2004). The Maximum Likelihood method was chosen as it allows for the estimation of all model path coefficients and to compute fit statistics. Also, to assess the overall model fit to empirical data, several goodness-of-fit measures and recommended cut-points were used: Chi-Square (χ^2^), Normed Chi-Square (χ^2^/df), Comparative Fit Index (CFI ≥ 0.95, desirable), Goodness of Fit Index (GFI ≥ 0.95, desirable), Root Mean Square Error of Approximation (RMSEA ≤ 0.05, good fit; ≤0.08, acceptable fit), with a 95% confidence interval (Hu and Bentler, 1998; Kline, 2005) and Standardized Root Mean Square Residual (SRMR good fit; ≤0.08; Hu and Bentler, 1998). The mediation effects were analyzed using a bootstrap procedure (2000 resamples) with a 95% bias-corrected confidence interval. It is considered that, if zero is not included in the interval between the lower and the upper bound, the effect is statistically significant at *p* < 0.05 (Kline, 2005).

## Results

### Preliminary descriptive and correlation analyses

The descriptive statistics and Pearson correlations for DDH duration, child’s age and psychological variables are presented in Table 1. Results showed that illness perception correlated positively and moderately with perceived stress and symptoms of anxiety and depression. Self-compassion and perceived social support correlated negatively with illness perception and psychological distress. The correlation between self-compassion and perceived social support was positive and of moderate magnitude. Finally, no significant correlations were found between DDH duration, child’s age and psychological variables.

**Table 1.** Descriptive statistics and correlations for medical, sociodemographic, and psychological variables.

Variable	*M* (SD)	1	2	3	4	5	6	7
1. Illness perception	35.48 (13.36)	—						
2. Self-compassion	36.16 (7.42)	−0.39**	—					
3. Perceived social support	5.82 (1.16)	−0.22*	0.47**	—				
4. Perceived stress	21.61 (7.61)	0.41**	−0.79**	−0.40**	—			
5. Anxiety	9.21 (3.76)	0.40**	−0.62**	−0.42**	0.74**	—		
6. Depressive symptoms	7.44 (4.25)	0.40**	−0.68**	−0.49**	0.67**	0.65**	—	
7. DDH duration	12.94 (15.72)	0.06	−0.05	−0.06	0.16	0.07	−0.01	—
8. Child’s age	17.80 (17.82)	0.07	−0.11	−0.08	0.18	0.13	0.05	0.87**

BRIEF-IPQ scores can range from 0 to 80 with higher scores indicating more threatening illness perceptions; SCS-SF scores range from 12 to 60; MSPSS mean scores range from 0 to 7; PSS-10 scores range from 0 to 40 and for each HADS subscale scores may range from 0 to 21. For all measures higher scores represent more of the construct being assessed.

* *p* < 0.05. **p < 0.001.

### Path analysis

**Figure 1.**
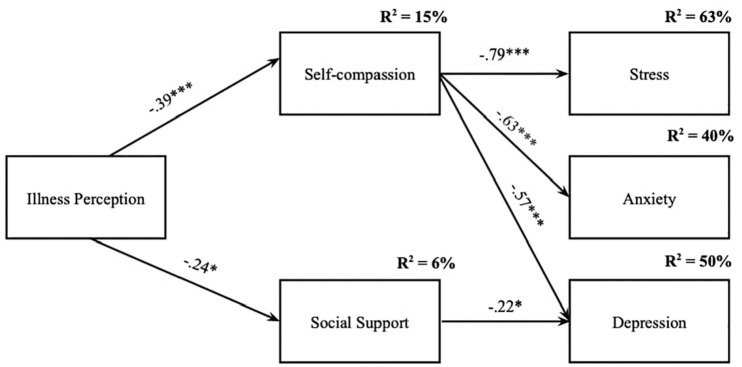
Final path model. Standardized path coefficients among variables are presented. All path coefficients are statistically significant. *p < 0.05. ***p < 0.001.

To test the mediator effect of self-compassion and social support on the relationship between mothers’ perception of their child’s DDH and psychological distress, a path analysis was performed. Firstly, a model was computed to ascertain the significance of the direct effects of illness perception (predictor) on the dependent measures (see Supplementary Material). Then, the initial mediation model was tested through a fully saturated model with 11 parameters. Model fit indices were neither examined nor reported as fully saturated models always have a perfect model fit. The analysis of the path coefficients from the first (fully saturated) model revealed that five path coefficients were not statistically significant and were progressively removed: social support → stress (*t*-statistics = 0.371; *p* = 0.517), social support → anxiety (*t*-statistics = −0.523; *p* = 0.146), illness perception → stress (*t*-statistics = 0.068; *p* = 0.087), illness perception → depression (*t*-statistics = 0.037; *p* = 0.126), illness perception → anxiety (*t*-statistics = 0.025; *p* = 0.231). The model was then respecified with all of the remaining individual path coefficients being statistically significant. The final model can be seen in Figure 1. Overall, the model presented a very good model fit to the data: χ^2^(5) = 11.147, *p* = 0.049; χ^2^/df = 2.229; GFI = 0.956; CFI = 0.977; RMSEA = 0.122; CI = 0.009, 0.220; *p* = 0.098; SRMR = 0.059.

The analysis of the total, direct and indirect associations showed only indirect effects of mothers’ perception of their child’s DDH on depression, anxiety, and stress symptoms. The indirect association of illness perception on stress through self-compassion was β = 0.307 (based on 95% CI: 0.152, 0.453, *p* = 0.001). The indirect effect on anxiety through self-compassion was β = 0.244 (based on 95% CI: 0.111, 0.384, *p* = 0.001). In addition, two positive and statistically significant indirect effects of illness perception on depression were found: one through self-compassion and another through social support (β = 0.272; based on 95% CI: 0.130, 0.409, *p* = 0.001). Overall, the final model (Figure 1) accounted for 15% of the variance in self-compassion, 6% in social support, 50% in depressive symptoms, 40% in anxiety, and 63% in perceived stress.

## Discussion

Facing a diagnosis of a child’s chronic illness may pose significant challenges for the psychological adjustment of parents (e.g. Gibbard et al., 2021). Previous research suggests the importance of social support and self-compassion to foster resilience and well-being and decrease depressive, anxiety, stress, and burnout symptoms in parents (Gerber et al., 2021; Toledano-Toledano and Luna, 2020). Thus, this study’s main goal was to test whether self-compassion and social support would mediate the relationship between mothers’ perception of their children’s DDH and their psychological adjustment.

As expected and consistent with previous studies (Cousineau et al., 2019; Cousino and Hazen, 2013; Toledano-Toledano and Luna, 2020), negative perceptions that mothers have of their child’s DDH were positively correlated with symptoms of stress, anxiety, and depression. Also, self-compassion and social support were negatively linked to these psychopathological symptoms. Thus, it seems that mothers with children diagnosed with DDH who have negative perceptions of their child’s DDH also displayed higher levels of psychopathological symptoms, reported less social support and less ability to be kind, supportive, and non-judgmental towards themselves when facing difficult times.

In addition, results from the path analysis suggested a mediating effect of self-compassion and social support on the relationship between mothers’ perception of their child’s DDH and the manifestation of psychological distress. The tested model accounted for 63%, 40%, and 50% of mothers’ symptoms of stress, anxiety, and depression, respectively. Among all the findings, some seem to be particularly interesting: first, the direct associations of mothers’ perception of their child’s DDH on psychological distress was mediated by self-compassion and social support by family, friends, and significant others. Also, the final model revealed that mothers’ perception of their child’s DDH association with stress, anxiety, and depression was mediated by the mothers’ self-compassion abilities. In contrast, social support seemed only to mediate the effect of mothers’ perception of their child’s DDH on depressive symptoms. Self-compassion has indeed been studied and shown to be an important antidote against psychological suffering and maladjustment in caregivers of chronically ill children (Cousineau et al., 2019; Gerber et al., 2021). Also, the perception of a child’s illness can be an additional and heavy challenge in parenting, that involves significant concerns about the child’s health and well-being (Cousineau et al., 2019). It can be associated with negative consequences for the mental health of parents (e.g. Cohn et al., 2020). Altogether, these findings suggest that approaching the suffering that may derive from a child’s illness in a self-compassionate way (i.e. by being aware of one’s suffering and experience in the present moment, understanding and framing suffering as a human and universal experience and directing kindness and understanding towards oneself), may reduce its impact on psychological distress. This is aligned with previous research with parents of children with CI that showed that self-compassion is associated with adaptive coping patterns in parents (Hawkins et al., 2019; Neff et al., 2018) and less tendency to withdraw from aversive situations and avoid its facts (Gerber and Anaki, 2019), being considered a promising avenue for pediatric clinical intervention (Brassington and Lomas, 2021; Germer and Neff, 2019). Recently, two intervention studies (Ahmed and Raj, 2023; Khosrobeigi et al., 2022) revealed that fostering self-compassion can promote positive outcomes and mitigate hopelessness and psychopathological symptoms in parents of children with CI. Self-compassion training helps parents stop criticizing themselves when dealing with difficult situations and have a warm and kind attitude towards themselves, which in turn enables them to take better care of their children (Ahmed and Raj, 2023).

Concerning perceived support from family, friends and close people, it seems that this may influence the relationship between mothers’ perceptions of the child’s DDH and depressive symptoms. Nevertheless, the amount of social support variance explained is low. Still, this result emphasizes the importance of feeling connected to others in a meaningful way in decreasing the likelihood of becoming depressed when facing a child’s chronic illness. This is aligned with other studies (Grey et al., 2020; Werner-Seidler et al., 2017) showing that social support is a key element to reduce the risk of depression and poorer sleep quality.

This finding further supports the idea that it is valuable to address parental needs, especially because of its link with an improvement in the quality of parenthood (Jacobs and Wachs, 2002; Roskam et al., 2017). In light of these results, it seems likely that an investment in addressing mothers’ suffering through the cultivation of self-compassion could diminish psychopathological symptoms, with potential benefits for mothers’ well-being and the quality of children’s care behaviors. For instance, brief compassion-focused interventions may be offered to all parents and used to screen for those who may need additional and more individualized support (Ahmed and Raj, 2023).

This study has nonetheless some limitations that should be considered. First, the sample is exclusively composed of mothers, which does not provide a fair representation of all parents. Nevertheless, we focus on mothers as they are still the primary caregivers of their children. Future studies should also pay attention to the perception and impact of the disease on fathers and other significant caregivers. Furthermore, the limited sample size and the online snowballing technique recruitment strategy are limitations. Recruitment through social media may prevent the determination of the response rate or the representativeness of the sample, which limits the interpretation of findings in a broader context. Another limitation refers to the cross-sectional design of the study, which limits inferences of causality. There is a need for studies of longitudinal nature, able to confirm the results obtained in this study. Moreover, the use of self-report questionnaires has limitations that could be overcome or complemented in the future by using structured or semi-structured interviews to better grasp mothers’ experiences and perceptions of their child’s DDH. Finally, the lack of objective measures of the severity of the children’s condition, length of time since diagnosis or the nature of treatment makes it difficult to characterize the sample, to determine its representativeness or to assess the degree to which objective medical variables explained any of the variance in the psychological findings.

In summary, this study showed that both self-compassion and perceived social support seem to be relevant to the psychological adjustment of mothers in the face of a child’s DDH. When this type of diagnosis is established, it is natural that the focus of mothers is mostly on the child and their treatments. Nevertheless, it is crucial to pay attention to how caregivers experience their adaptation to this reality. It has been previously suggested (Gérain and Zech, 2018) that parental coping skills should be included as part of medical care. Our study contributes to the existing literature by highlighting the importance of inter and intrapersonal factors, namely social support and self-compassion abilities, in mothers’ psychological adjustment to a child’s DDH. It is essential to assess the protection and adjustment skills of these mothers and, if needed, encourage them to seek social support, but also their abilities for self-compassion, particularly when facing the challenges of caring for a child with DDH.

## Supplemental Material

sj-docx-1-hpq-10.1177_13591053241295892 – Supplemental material for The roles of self-compassion and social support on the maternal adjustment to a child’s hip dysplasia Supplemental material, sj-docx-1-hpq-10.1177_13591053241295892 for The roles of self-compassion and social support on the maternal adjustment to a child’s hip dysplasia by Bruna Veloso, Lara Palmeira, Lénia Carvalhais, Joana Marta-Simões and Inês A A Trindade in Journal of Health Psychology
