# Supplementary material for: The roles of self-compassion and social support on the maternal adjustment to a child’s hip dysplasia
Source: J Health Psychol. 2024 Nov 21;30(9):2172–82. doi: 10.1177/13591053241295892 (PMC12322331; doi:10.1177/13591053241295892)
Supplement: sj-docx-1-hpq-10.1177_13591053241295892 – Supplemental material for The roles of self-compassion and social support on the maternal adjustment to a child’s hip dysplasia [file sj-docx-1-hpq-10.1177_13591053241295892.docx]

**Supplementary material**

Path model of the direct effect of mothers’ illness perception on the dependent measures (see Figure 1). Results showed that all direct effects were statistically significant. The direct effect of illness perception on stress was β = .412 (based on 95% CI: .218; .577, *p* = .001), direct effect of illness perception on anxiety was β = .416 (based on 95% CI: .222; .579, *p* = .001), and direct effect of illness perception on depression was β = .408 (based on 95% CI: .220; .570, *p* = .001).

The model accounted for 16,9% of stress, 17,3% of anxiety and 16,6% of depressive symptoms variance.

**Figure 1 Supplementary material**

*Path Model of the direct effect of illness perception on mothers' psychopathological symptoms*

*
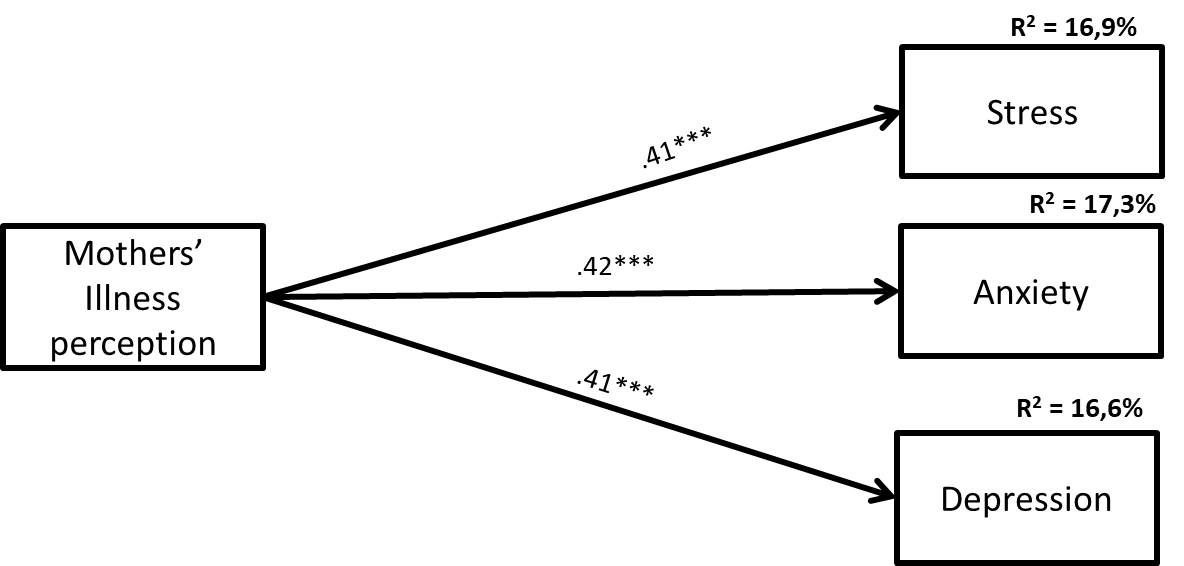
*

Note. ****p* < .001; Standardized path coefficients among variables are presented. All path coefficients are statistically significant.
